# Opportunities for understanding the COVID-19 pandemic and child health in the United States: the Environmental influences on Child Health Outcomes (ECHO) program

**DOI:** 10.3389/fped.2023.1171214

**Published:** 2023-06-15

**Authors:** Traci A. Bekelman, Leonardo Trasande, Andrew Law, Courtney K. Blackwell, Lisa P. Jacobson, Theresa M. Bastain, Carrie V. Breton, Amy J. Elliott, Assiamira Ferrara, Margaret R. Karagas, Judy L. Aschner, Nicole Bornkamp, Carlos A. Camargo, Sarah S. Comstock, Anne L. Dunlop, Jody M. Ganiban, James E. Gern, Catherine J. Karr, Rachel S. Kelly, Kristen Lyall, T. Michael O’Shea, Julie B. Schweitzer, Kaja Z. LeWinn

**Affiliations:** ^1^ Lifecourse Epidemiology of Adiposity and Diabetes (LEAD) Center, University of Colorado Anschutz Medical Campus, Aurora, CO, United States; ^2^ Department of Pediatrics, Department of Environmental Medicine, New York University Grossman School of Medicine, New York, NY, United States; ^3^ Department of Epidemiology, Johns Hopkins Bloomberg School of Public Health, Baltimore, MD, United States; ^4^ Department of Medical Social Sciences, Feinberg School of Medicine, Northwestern University, Chicago, IL, United States; ^5^ Department of Population and Public Health Sciences, Keck School of Medicine, University of Southern California, Los Angeles, CA, United States; ^6^ Avera Research Institute, Department of Pediatrics, University of South Dakota School of Medicine, Sioux Falls, SD, United States; ^7^ Division of Research, Kaiser Permanente Northern California, Oakland, CA, United States; ^8^ Department of Epidemiology, Geisel School of Medicine at Dartmouth, Hanover, NH, United States; ^9^ Departments of Pediatrics, Albert Einstein College of Medicine, Bronx, NY, United States; ^10^ Department of Pediatrics, Hackensack Meridian School of Medicine, Nutley, NJ, United States; ^11^ Division of Chronic Disease Research Across the Lifecourse, Department of Population Medicine, Harvard Medical School and Harvard Pilgrim Health Care Institute, Boston, MA, United States; ^12^ Department of Emergency Medicine, Massachusetts General Hospital, Harvard Medical School, Boston, MA, United States; ^13^ Department of Food Science and Human Nutrition, Michigan State University, East Lansing, MI, United States; ^14^ Department of Gynecology and Obstetrics, Emory University School of Medicine, Atlanta, GA, United States; ^15^ Department of Psychological and Behavioral Sciences, The George Washington University, Washington, DC, United States; ^16^ Department of Pediatrics, University of Wisconsin School of Medicine and Public Health, Madison, WI, United States; ^17^ Departments of Pediatrics & Occupational and Environmental Health Sciences, University of Washington, Seattle, WA, United States; ^18^ Channing Division of Network Medicine, Brigham and Women’s Hospital and Harvard Medical School, Boston, MA, United States; ^19^ AJ Drexel Autism Institute, Drexel University, Philadelphia, PA, United States; ^20^ Department of Pediatrics, University of North Carolina School of Medicine, Chapel Hill, NC, United States; ^21^ Department of Psychiatry and Behavioral Sciences, University of California Davis School of Medicine, Sacramento, CA, United States; ^22^ Department of Psychiatry and Behavioral Sciences, University of California, San Francisco, San Francisco, CA, United States

**Keywords:** life course approach, environmental exposures, health disparities, parent-child dyads, pediatric health, health behaviors

## Abstract

**Objective:**

Ongoing pediatric cohort studies offer opportunities to investigate the impact of the COVID-19 pandemic on children's health. With well-characterized data from tens of thousands of US children, the Environmental influences on Child Health Outcomes (ECHO) Program offers such an opportunity.

**Methods:**

ECHO enrolled children and their caregivers from community- and clinic-based pediatric cohort studies. Extant data from each of the cohorts were pooled and harmonized. In 2019, cohorts began collecting data under a common protocol, and data collection is ongoing with a focus on early life environmental exposures and five child health domains: birth outcomes, neurodevelopment, obesity, respiratory, and positive health. In April of 2020, ECHO began collecting a questionnaire designed to assess COVID-19 infection and the pandemic's impact on families. We describe and summarize the characteristics of children who participated in the ECHO Program during the COVID-19 pandemic and novel opportunities for scientific advancement.

**Results:**

This sample (*n *= 13,725) was diverse by child age (31% early childhood, 41% middle childhood, and 16% adolescence up to age 21), sex (49% female), race (64% White, 15% Black, 3% Asian, 2% American Indian or Alaska Native, <1% Native Hawaiian or Pacific Islander, 10% Multiple race and 2% Other race), Hispanic ethnicity (22% Hispanic), and were similarly distributed across the four United States Census regions and Puerto Rico.

**Conclusion:**

ECHO data collected during the pandemic can be used to conduct solution-oriented research to inform the development of programs and policies to support child health during the pandemic and in the post-pandemic era.

## Introduction

Research studies are needed to understand whether and how the social and economic disruption of the COVID-19 pandemic and/or SARS-CoV-2 infection affected the health of children in the United States (US) (1). Environmental exposures, health behaviors, and health status during the pandemic may have implications for children's future health, especially if environmental exposures or health behaviors altered by the pandemic persist in the post-pandemic era (e.g., parent unemployment, shuttered community resources, new modes of physical activity) (2–4). Moreover, pandemic-related exposures (e.g., food insecurity, parent illness) occurring during developmentally sensitive periods (5) may have implications for health across the life course, even if the exposures are time-limited (6).

As of September 2022, there were over 300,000 COVID-19-related publications in PubMed, yet only a small proportion (around 10% or less) are focused on children's health. There is evidence of both adverse and favorable changes in children's environments, behaviors, and health status during the pandemic (7, 8). However, there are concerns about the quality of the evidence and representativeness of study samples. Most studies that included primary data collection in children during the pandemic were conducted outside the US (9–14). Early studies conducted among US children provided some insights into whether the social and economic disruption of the pandemic had implications for children's health status and behaviors, with a focus on the initial lockdown stage (15, 16). However, most studies were conducted among small and homogenous samples, and had methodological limitations, such as the use of unvalidated questionnaires to assess health outcomes or cross-sectional study designs with a risk of recall bias (17, 18). While more recent work has capitalized on national samples in the US, such as the Adolescent Brain and Cognitive Development (ABCD) cohort (19–23), there is still a relative dearth of high-quality studies that include children at all stages of development.

The data sources needed to conduct these types of high-quality analyses on pediatric health and the COVID-19 pandemic are limited because public health precautions, such as physical distancing requirements, or hesitancy to attend in-person research visits among potential study participants, reduced the feasibility of conducting primary data collection during the pandemic. For example, field operations were halted during the pandemic for large national surveys that had planned to assess variables relevant for child health in 2020 and 2021. This included the National Health and Nutrition Examination Survey (NHANES) and the American Community Survey, conducted by the Centers for Disease Control and Prevention and the US Census Bureau, respectively (24, 25). This is concerning because the cessation of national studies that include diverse samples of children in regard to race, ethnicity, and socioeconomic status potentially created a gap in understanding of inequities in the COVID-19 experience (26, 27).

In September 2016, the National Institutes of Health (NIH) launched a 7-year initiative, the Environmental influences on Child Health Outcomes (ECHO) Program (28). ECHO is a national consortium of 69 new and established pregnancy and pediatric cohort studies. The consortium was designed to be a large, population-based cohort of U.S. children unified under a single research protocol. Primary data collection in the ECHO Program, using this standardized protocol among US children and their caregivers, not only continued during the pandemic in-person, as allowed, but also pivoted to remote data collection methods (eg, remote informed consent procedures and collection of biospecimens such as blood spots, urine, and hair samples; online surveys for self-administration; phone-based questionnaires; and administrator-assisted anthropometric assessments via video conferencing). The ECHO Program also expanded to include time-sensitive assessments of COVID-19 infection and pandemic-related psychosocial impacts, thus providing a valuable data source for understanding whether and how the social and economic disruption of the COVID-19 pandemic and/or SARS-CoV-2 infection affected the health of US children. As part of this effort, the ECHO Program conducted rapid response research via COVID-19 Administrative Supplements funded by the NIH, with a focus on using existing and novel tools. Additionally, the ECHO Program fostered innovation in measurement by developing and administering novel, publicly available COVID-19-specific questionnaires for caregivers and children (29).

The objective of this research brief is to provide an overview of what the ECHO Program is uniquely poised to contribute to our understanding of the COVID-19 pandemic and child health. Specifically, we propose to (1) inform the scientific community about the characteristics of parent-child dyads who participated in data collection as part of the ECHO Program during the COVID-19 pandemic, and (2) describe the types of innovative research questions that can be answered with those data, which will be de-identified and publicly available in 2023.

## Methods

The ECHO Program aims to examine the effects of physical, chemical, social, behavioral, biological, natural, and built environmental exposures on five key child health outcomes: pre-, peri-, and postnatal; upper and lower airway; obesity; neurodevelopment; and positive health (http://echochildren.org) (30). The ECHO Cohort is described in detail here (31). Briefly, ECHO enrolls children and their caregivers from community- and clinic-based studies. New data were collected under a standardized data collection protocol, the ECHO-Wide Cohort Protocol (EWCP), beginning in 2019 and continuing through 2023. Extant data from each of the pediatric cohorts collected prior to the inception of the EWCP were pooled and harmonized at the centralized ECHO Data Analysis Center at Johns Hopkins University and RTI International. Institutional review boards monitored human subject activities at each cohort site and the Data Analysis Center. Caregivers provided informed consent, and children provided assent or consent, as appropriate. Many cohorts offered an “e-consent” option during the pandemic, in which the informed consent process was conducted via video conference. Additionally, RedCap is a secure, HIPAA- and FISMA-compliant web platform for building and managing online surveys and databases. The ECHO Program encourages the use of RedCap Central, a centralized version of RedCap. The purpose of RedCap Central is to facilitate immediate and automatic transfer of data from the ECHO Cohorts to the centralized ECHO Data Analysis Center to be used in pooled analyses and ECHO-wide scientific publications. De-identified data collected with the EWCP during the COVID-19 pandemic, along with selected extant data collected pre-pandemic, will be available to the public in 2023 via the Eunice Kennedy Shriver National Institute of Child Health and Human Development (NICHD) Data and Specimen Hub (DASH) (https://dash.nichd.nih.gov/) (32). In addition to the data themselves, the publicly available DASH website includes downloadable versions of the full ECHO data collection protocol, data collection forms, data dictionaries and a detailed description of study methodologies. DASH is designed to enhance the accessibility, and expand the reach, of ECHO data to provide opportunities for researchers worldwide to answer important questions about child health.

In addition to ongoing data collection with the EWCP, a novel ECHO COVID-19 questionnaire was developed in April 2020 in English and Spanish for the ECHO Program (29). The questionnaire included three versions: caregiver self-report, adolescent self-report and caregiver-report on child for children 12 years and younger. The original questionnaire was designed to assess SARS-CoV-2 infection, access to health-related services, impact on employment, changes in health behaviors, and the psychosocial impact of the pandemic on caregivers and children (e.g., pandemic-related parent and child stress, coping mechanisms, social connectedness). The ECHO Program leveraged existing infrastructure to quickly mobilize the collection of this new questionnaire, which was subsequently modified in later phases of the pandemic to include additional factors as the pandemic evolved, such as vaccine administration, vaccine hesitancy, and remote schooling.

To summarize the data available, we report characteristics of children from birth to 21 years who participated in the EWCP during the first 17 months of the pandemic. The number of children who completed the protocol and the ECHO COVID-19 questionnaire is reported for the full 17 months (4/1/2020–8/31/2021) and by time period: Period 1 (4/1/2020–5/31/2020), Period 2 (6/1/2020–8/31/2020), Period 3 (9/1/2020–5/31/2021, “2020/21 academic school year”) and Period 4 (6/1/2021–8/31/2021), by age group, child sex, race, ethnicity, level of maternal education and region of residence. The World Health Organization declared COVID-19 a pandemic on March 11, 2020, and the ECHO Program amended its protocol to incorporate the COVID-19 questionnaires in April 2020. April 1 was used as the start date for data described in this paper because the ECHO questionnaires queried behaviors in the preceding week to month. The time periods were selected to generally align with the school year and summer vacation, although there is some variation in the start and end dates of the academic year by school district.

## Results

**Figure 1:**
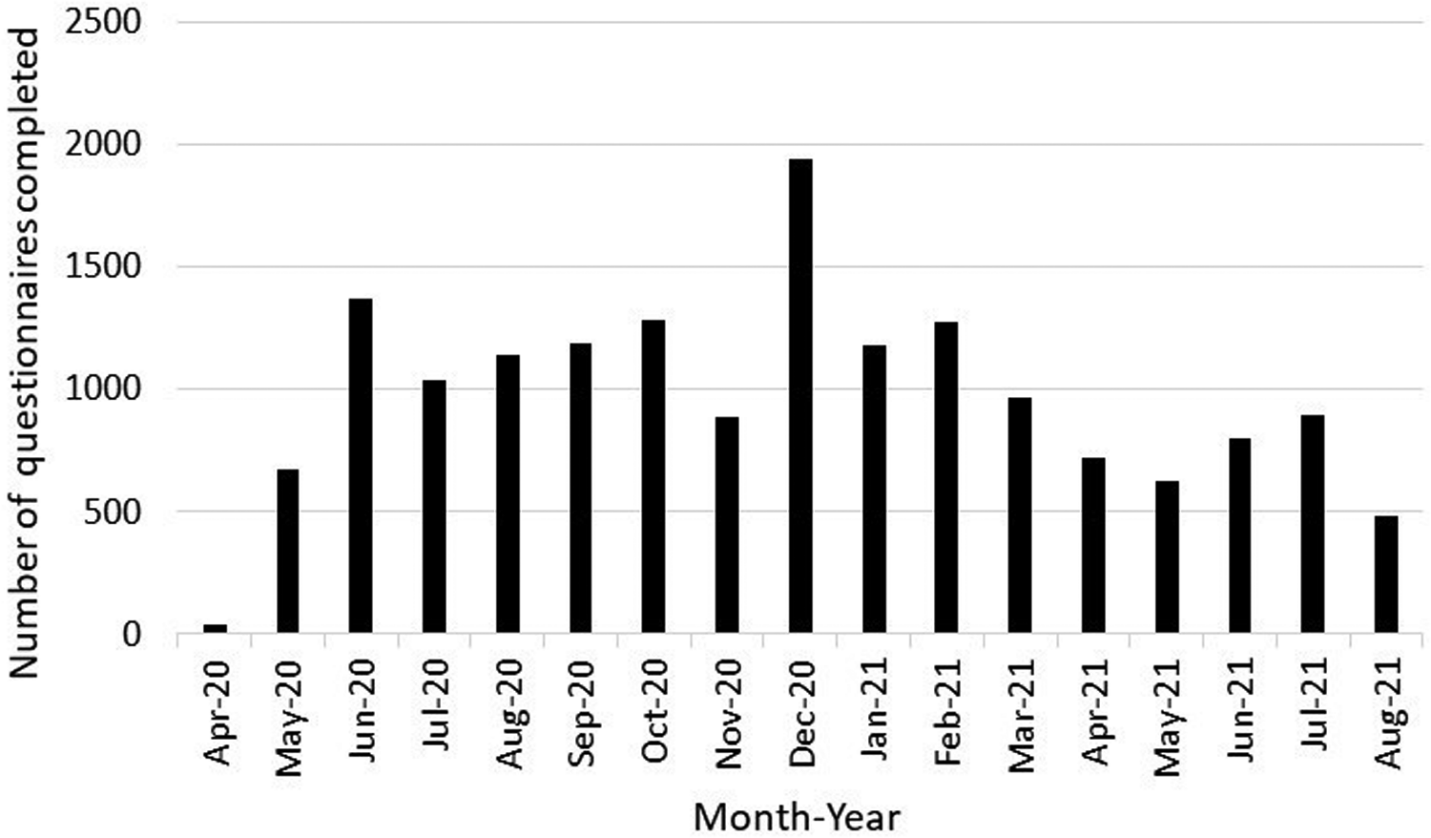
Number of COVID-19 questionnaires administered to children birth to 21 years and their caregivers who participated in the ECHO-wide common protocol during the first 17 months of the COVID-19 pandemic by month of administration.

Characteristics of children from birth to age 21 years who participated in the ECHO-Wide Cohort Protocol during the first 17 months of the pandemic and completed a COVID-19 questionnaire are shown in Table 1. Sixty cohorts contributed data from 13,725 children between April 1, 2020 and August 31, 2021. Participants were included from multiple life stages: early childhood (31%), middle childhood (41%), and adolescence up to age 21 (16%). The lower proportion of participants from the adolescent life stage compared to younger life stages is consistent with overall ECHO Cohort, and likely does not represent a selection bias. The pediatric sample was 49% female, 22% Hispanic, and children were similarly distributed across the four United States Census regions and Puerto Rico. Child race was 64% White, 15% Black, 3% Asian, 2% American Indian or Alaska Native, <1% Native Hawaiian or Pacific Islander, 10% Multiple race and 2% Other race. Fifty-seven percent of caregivers had a bachelor's degree or higher. Most data were collected during the 2020–2021 academic school year (*n* = 10,058), followed by Summer 2020 (*n* = 3,548), Summer 2021 (*n* = 2,180), and the first two months of the pandemic (*n* = 708). The lower number of cohorts administering COVID-19 questionnaires in the first two months of the pandemic (*n* = 21 cohorts) compared to the other time Periods (*n* ≥ 50 cohorts) reflects the staggered timing across the US when clinical research units were permitted to resume data collection. Nineteen percent of participants completed more than one COVID-19 questionnaire during the 17-month period. As shown in Figure 1, COVID-19 questionnaires were administered during different phases of the pandemic (e.g., the initial lockdown period, as well as periods of less restrictive public health precautions), all seasons of the year, during both the academic school year and school vacations, and before and after COVID-19 vaccines became available to adults and youth.

**Table 1:** Characteristics of children birth to 21 years who participated in the ECHO-wide cohort protocol during the first 17 months of the COVID-19 pandemic and completed an ECHO COVID-19 questionnaire *via* self-report or caregiver-proxy report.

Sample characteristics	Full sample	Period 1	Period 2	Period 3	Period 4
	(4/1/2020–8/31/2021)	(4/1/2020–5/31/2020)	(6/1/2020–8/31/2020)	(9/1/2020–5/31/2021)	(6/1/2021–8/31/2021)
Number of cohorts contributing data	60	21	53	57	50
Number of children	13,725	709	3,548	10,058	2,180
Child age at COVID-19 survey completion, *n* (%)^a^	–	709 (100.0)	3,548 (100.0)	10,058 (100.0)	<2,185 (100.0)
Birth-24 months	–	0 (0.0)	0 (0.0)	17 (<1.0)	<5 (<1.0)
2–5 years	–	126 (17.8)	1,013 (28.6)	2,954 (29.4)	649 (29.8)
6–12 years	–	221 (31.2)	1,526 (43.0)	4,206 (41.8)	1,066 (48.9)
13–16 years	–	65 (9.2)	260 (7.3)	633 (6.3)	131 (6.0)
17–21 years	–	144 (20.3)	303 (8.5)	841 (8.4)	92 (4.2)
Child sex assigned at birth, *n* (%)	13,716 (99.0)	708 (99.0)	3,548 (100.0)	10,052 (99.0)	2,178 (99.0)
Male	6,948 (50.6)	357 (50.4)	1,773 (50.0)	5,066 (50.4)	1,107 (50.8)
Female	6,768 (49.3)	351 (49.5)	1,775 (50.0)	4,986 (49.6)	1,071 (49.1)
Race, *n* (%)	<13,060 (95.1)	<680 (94.9)	3,372 (95.04)	9,619 (95.6)	<2,090 (95.5)
White	8,739 (63.7)	392 (55.3)	2,455 (69.2)	6,620 (65.8)	1,431 (65.6)
Black	2,007 (14.6)	132 (18.6)	386 (10.9)	1,371 (13.6)	272 (12.5)
Asian	432 (3.2)	16 (2.3)	88 (2.5)	306 (3.0)	67 (3.1)
Native Hawaiian or other Pacific Islander	<30 (<1.0)	<5 (<1.0)	5 (<1.0)	21 (<1.0)	<5 (<1.0)
American Indian or Alaska Native	281 (2.1)	6 (<1.0)	102 (2.9)	224 (2.2)	82 (3.8)
Multiple Race	1,351 (9.8)	86 (12.1)	304 (8.6)	939 (9.3)	205 (9.4)
Other Race	218 (1.6)	39 (5.5)	32 (<1.0)	138 (1.4)	24 (1.1)
Ethnicity, *n* (%)	13,594 (99)	699 (98.6)	3,530 (99.0)	9,973 (99.0)	2,144 (98.4)
Hispanic	3,056 (22.27)	201 (28.4)	626 (17.6)	2,102 (20.9)	411 (18.9)
Not of Hispanic, Latino or Spanish origin	10,538 (76.8)	498 (70.2)	2,904 (81.9)	7,871 (78.3)	1,733 (79.5)
Level of formal caregiver education, *n* (%)	13,207 (96.2)	694 (97.9)	3,376 (95.2)	9,690 (96.3)	2,133 (97.8)
Less than high school degree	643 (4.7)	36 (5.1)	149 (4.2)	440 (4.4)	95 (4.4)
High school degree, GED or equivalent	1,483 (10.8)	103 (14.5)	355 (10.0)	1,064 (10.6)	189 (8.7)
Some college, no degree; Associate's degree; Trade school	3,266 (23.8)	178 (25.1)	830 (23.4)	2,320 (23.1)	567 (26.0)
Bachelor's degree and above	7,815 (56.94)	377 (53.2)	2,042 (57.6)	5,866 (58.3)	1,282 (58.8)
Region of residence, *n* (%)	13,725 (100.0)	709 (100.0)	3,548 (100.0)	10,058 (100.0)	2,180 (100.0)
West	3,216 (23.4)	93 (13.1)	546 (15.4)	2,281 (22.7)	688 (31.6)
Midwest	3,377 (24.6)	127 (17.9)	973 (27.4)	2,579 (25.6)	773 (35.4)
South	3,008 (21.9)	70 (9.9)	541 (15.3)	2,237 (22.2)	309 (14.2)
Northeast	4,124 (30.1)	419 (59.1)	1,488 (41.9)	2,961 (29.4)	410 (18.8)

^a^ Birth-5 years = early childhood; 6–12 years = middle childhood; 13–21 years = adolescence. We did not include numbers for the full sample by age group because some children passed through multiple age groups during the study period and completed questionnaires at more than one age group.

## Discussion

During a period of unprecedented public health disruptions and precautions in the US, the ECHO Program examined COVID-19-related environmental conditions and health outcomes among 13,725 socioeconomically and racially diverse children and their caregivers. This included a standardized data collection protocol that was initially implemented pre-pandemic, as well as an expanded protocol to include novel approaches to assessing pandemic-related exposures and outcomes. Strengths of these data include the large sample size and diversity by child age, sex, race, Hispanic ethnicity, and US region of residence. The high proportion of caregivers with a Bachelor's degree (57%) suggests that this sample may not be representative of the general population (38% in general US) (33), but this limitation is attenuated by the large number of caregiver-child dyads who participated (*n* = 13,723) and heterogeneity (e.g., over 5,000 caregivers who participated in ECHO during the pandemic did not have a Bachelor's degree) facilitating robust analyses of socioeconomic issues.

Future publications can leverage these richly characterized data from the ECHO Program to: (1) Examine the short- and long-term impacts of SARS-CoV-2 infection among pregnant mothers and children on children's birth outcomes, airways, obesity, neurodevelopment, and positive health using harmonized data collected before and during the pandemic; (2) Evaluate societal changes during the pandemic and their effects on the five child health outcomes which are a focus of ECHO; (3) Follow COVID-infected subpopulations for other subclinical effects of infection and/or other pandemic-related exposures that may be revealed through biospecimen assays or quantification of other biomarkers; (4) Compare a broad range of physical, chemical, social, behavioral, biological, natural, and built environmental exposures and health outcomes before and during the pandemic; (5) Provide insight into whether and how societal changes associated with the pandemic may differentially impact the health of children from different socioeconomic, racial, and ethnic groups and, in turn, exacerbate existing health inequities; (6) Describe geographic variation in environmental exposures and health outcomes during the pandemic using geocoded data, and describe how geographic location may modify the association between pandemic-related exposures and child health; (7) Identify early life factors associated with health outcomes during the pandemic, and related indicators of resilience and susceptibility, using a life course approach and harmonized extant data; and (8) Characterize the prevalence, disparities, and risk factors for SARS-CoV-2 infection among children. The ECHO Program has already begun leveraging these data to describe families' experiences during the COVID-19 pandemic, and the impact of the pandemic on the health (34–42), but many innovative research questions remain to be answered.

Importantly, ECHO data collected during the pandemic can be used to conduct solution-oriented research to inform the development of programs and policies that are customized to the “new normal” in the post-pandemic era. Societal changes spurred by the pandemic (e.g., caregiver remote working), and some economic impacts of the pandemic (e.g., shuttered community resources) persist, even as the number and proportions of vaccinated persons increases and public health precautions are lifted (43). The ECHO Program offers the unique opportunity to leverage well-characterized data from the largest ongoing pediatric multi-cohort research consortia in the US to understand the impact of the COVID-19 pandemic on child health.

## Data Availability

The data analyzed in this study are subject to the following licenses/restrictions: De-identified data from the ECHO Program are available through NICHD's Data and Specimen Hub (DASH). DASH is a centralized resource that allows researchers to access data from various studies via a controlled-access mechanism. Researchers can now request access to these data by creating a DASH account and submitting a Data Request Form. The NICHD DASH Data Access Committee will review the request and provide a response in approximately two to three weeks. Once granted access, researchers will be able to use the data for three years. See the DASH Tutorial for more detailed information on the process. Requests to access these datasets should be directed to https://dash.nichd.nih.gov/.
